# Advances in pancreatic islet monolayer culture on glass surfaces enable super-resolution microscopy and insights into beta cell ciliogenesis and proliferation

**DOI:** 10.1038/srep45961

**Published:** 2017-04-12

**Authors:** Edward A. Phelps, Chiara Cianciaruso, Jaime Santo-Domingo, Miriella Pasquier, Gabriele Galliverti, Lorenzo Piemonti, Ekaterine Berishvili, Olivier Burri, Andreas Wiederkehr, Jeffrey A. Hubbell, Steinunn Baekkeskov

**Affiliations:** 1 Institute of Bioengineering, School of Life Sciences, École Polytechnique Fédérale de Lausanne, CH-1015 Lausanne, Switzerland; 2 Graduate Program in Biotechnology and Bioengineering, School of Life Sciences, École Polytechnique Fédérale de Lausanne, CH-1015 Lausanne, Switzerland; 3 Nestlé Institute of Health Sciences S.A., EPFL Innovation Park, CH-1015 Lausanne, Switzerland; 4 Swiss Institute for Experimental Cancer Research (ISREC), School of Life Sciences, École Polytechnique Fédérale de Lausanne, CH-1015 Lausanne, Switzerland; 5 Pancreatic Islet Processing Facility, Diabetes Research Institute, IRCCS San Raffaele Scientific Institute, 20132 Milan, Italy; 6 Cell Isolation and Transplantation Center, Faculty of Medicine, Department of Surgery, Geneva University Hospitals and University of Geneva, CH-1211 Geneva, Switzerland; 7 BioImaging and Optics Core Facility, School of Life Sciences, École Polytechnique Fédérale de Lausanne, CH-1015 Lausanne, Switzerland; 8 Institute for Molecular Engineering, University of Chicago, Chicago, IL 60615, USA

## Abstract

A robust and reproducible method for culturing monolayers of adherent and well-spread primary islet cells on glass coverslips is required for detailed imaging studies by super-resolution and live-cell microscopy. Guided by an observation that dispersed islet cells spread and adhere well on glass surfaces in neuronal co-culture and form a monolayer of connected cells, we demonstrate that in the absence of neurons, well-defined surface coatings combined with components of neuronal culture media collectively support robust attachment and growth of primary human or rat islet cells as monolayers on glass surfaces. The islet cell monolayer cultures on glass stably maintain distinct mono-hormonal insulin+, glucagon+, somatostatin+ and PP+ cells and glucose-responsive synchronized calcium signaling as well as expression of the transcription factors Pdx-1 and NKX-6.1 in beta cells. This technical advance enabled detailed observation of sub-cellular processes in primary human and rat beta cells by super-resolution microscopy. The protocol is envisaged to have broad applicability to sophisticated analyses of pancreatic islet cells that reveal new biological insights, as demonstrated by the identification of an *in vitro* protocol that markedly increases proliferation of primary beta cells and is associated with a reduction in ciliated, ostensibly proliferation-suppressed beta cells.

The culture of cells on glass surfaces is an important step in sample preparation for high-resolution imaging of molecules within live and fixed cells. Imaging targets in close proximity to the surface of glass coverslips is a general requirement for visualization with high power objectives. Super-resolution microscopy techniques^1^ such as stimulated emission depletion (STED) microscopy, which permit the sub-diffraction limit discernment of biological structures, require cells to be grown on 0.17 mm glass coverslips for optimal performance and are incompatible with plastic substrates. The field of pancreatic islet biology has been hampered in the ability to utilize such advanced sub-cellular imaging techniques due to difficulties in culturing dissociated primary islet cells on the surface of glass coverslips^2^,^3^,^4^. Cell lines derived from pancreatic endocrine tumors, such as the beta cell lines INS-1^5^ and MIN6^6^, are amenable for culturing on glass. However, these cell lines fail to correctly recapitulate many key characteristics of primary beta cells and the cultures lack the signaling community of islet non-beta endocrine cells^7^. While existing techniques permit the culture of primary islet cell monolayers on tissue culture plastic, these methods perform sub-optimally in terms of adhesion and spreading when applied to glass surfaces.

Methods which successfully establish two-dimensional cultures of dispersed primary islet cells on plastic, include coating of the plastic surface with extracellular matrix (ECM) secreted from 804 G rat bladder carcinoma cells^3^,^4^,^8^, HTB-9 human bladder carcinoma cells^4^,^9^,^10^, A-431 human epidermoid carcinoma cells^11^, or bovine corneal epithelial cell matrix (BCEM)^4^,^11^,^12^. In our experience, these matrices promote adequate islet cell adhesion on tissue-culture plastics^12^. However, they result in suboptimal spreading and adhesion of primary islet cells on glass. Furthermore, cell-line derived ECMs have a high batch-to-batch variability that affects reproducibility and results in rapid de-differentiation of beta cells during monolayer culture^10^,^13^,^14^,^15^,^16^, highlighting the need to establish alternative culture techniques that better maintain differentiated islet cell phenotypes. The development of a robust and reproducible method for culturing monolayers of differentiated primary islet cells on glass would facilitate detailed imaging of subcellular processes such as insulin granule dynamics^17^,^18^, calcium signaling^19^, mitochondrial function^20^, or cytoskeletal morphology^21^,^22^.

In this study, we show that defined surface coatings of purified collagen IV or laminin combined with a cell culture medium originally formulated for primary neurons, promote superior adhesion, spreading and viability of human and rat islet cell monolayers while retaining key features of differentiated islet endocrine cells as well as beta cell function. Analyses of such monolayer cultures of primary islet cells on glass by high resolution microscopy enabled visualization of cilia morphology in primary beta cells, and the identification of a mechanistic correlation between disassembly of primary cilia and stimulation of beta cell proliferation.

Mature islet beta cells are typically quiescent, and methods that promote proliferation and expansion of beta cell mass are needed for research and as potential sources of beta cell replacement therapy. We illustrate below the use of our new monolayer culture system to identify conditions that markedly enhance primary rat beta cell proliferation. In addition to depending on mitogenic growth and survival factors, the proliferation of beta cells is suspected to be governed by the expression of primary cilia. The primary cilium is a microtubule-based structure projecting from the mother centriole during the G1/G0 phase of the cell cycle^23^. The primary cilium and the mitotic spindle both originate from the same structure, the centriole, and cannot exist simultaneously in most cell types^23^,^24^. As such, cilia may serve as proliferation suppressors that contribute to maintenance of cellular quiescence and homeostasis. Ciliogenesis and cell proliferation are bi-directionally regulated through a variety of signaling pathways such as those involving Wnt and beta-catenin, while defects in cilia signaling contribute to proliferative disorders^25^. Notably, pancreatic islet endocrine cells express a protruding primary cilium^26^. Intriguingly, factors reported to induce proliferation of insulin-producing beta cells such as increased Wnt/beta-catenin signaling^24^,^28^, Rho Kinase (ROCK) inhibition^28^ and overexpression of Aurora Kinase A^28^ are also implicated in pathways that limit primary cilia. Given this potential interconnection between beta-cell growth stimulatory factors and ostensibly necessary reduction in growth-suppressing cilia, we characterized cilia in our islet cell monolayer culture in the context of inducing proliferation of primary beta cells whilst maintaining their authenticity.

## Results

### Co-culture of primary islet cells with primary hippocampal neurons promotes islet cell adhesion on glass coverslips

**Figure 1:**
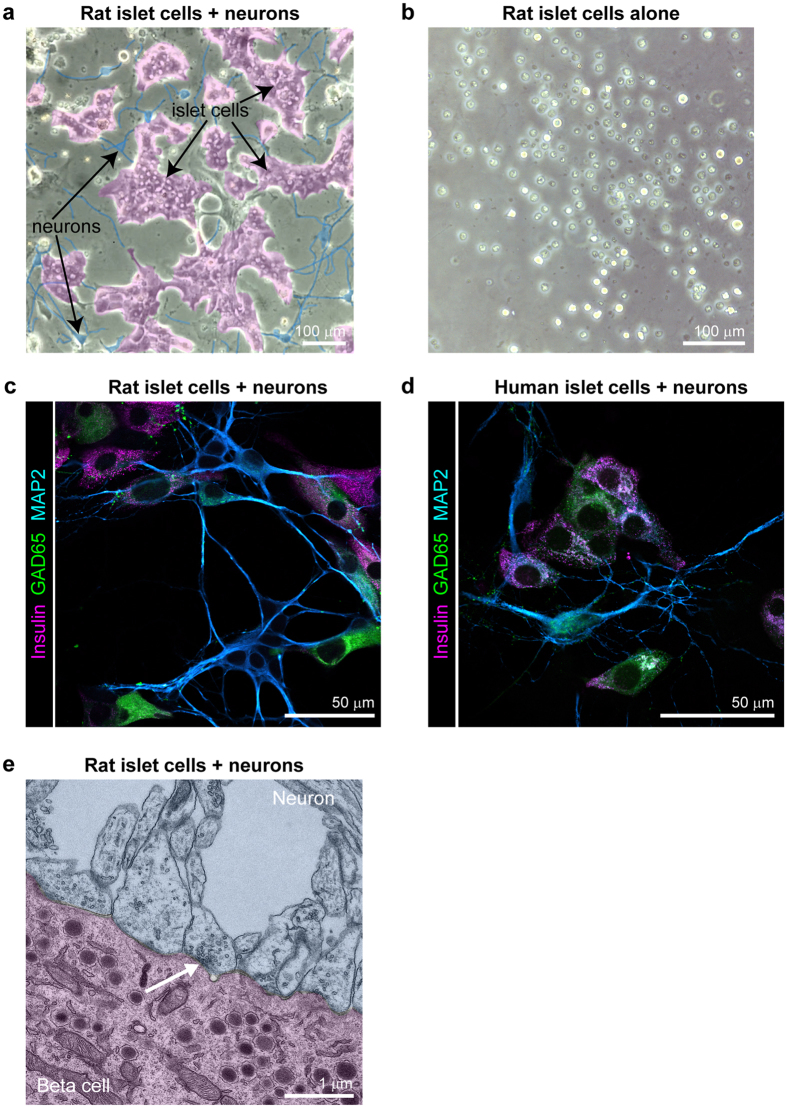
Neuron and rat islet cell co-culture. (**a**) A representative transmitted light image of rat islet cells (magenta) co-cultured with rat neurons (blue) on polyornithine-coated coverslips in neuronal medium, pseudocoloring added to highlight cell types identified by morphology. (**b**) A Representative transmitted light image of rat islet cells seeded alone on polyornithine-coated coverslips in neuronal medium. (**c**) Co-culture of neurons and rat islet cells immunostained for insulin expressed in beta cells, GAD65 expressed in both beta cells and in the soma and axons of GABA-ergic neurons, and MAP2 expressed in dendrites of all neurons. (**d**) Co-culture of neurons and human islet cells immunostained for insulin, expressed in beta cells, GAD65, expressed in beta cells and in the soma and axons of GABA-ergic neurons, and MAP2, expressed in dendrites of all neuronal cells. (**e**) TEM image of rat islet cells co-cultured with rat neurons showing synapse-like cell junctions (arrow) between a neuron (blue) containing synaptic vesicles and a beta cell (magenta) containing insulin granules, pseudo-coloring added to highlight cell types.

Pancreatic islets are innervated by the autonomic nervous system^30^,^31^, an observation that together with the expression of neurotransmitter receptors and neurotransmitters in islet cells^32^ has long implicated neuronal signaling in islet function. Intrigued by the neuroendocrine character of islet cells, we asked whether co-culture with primary neurons influenced the growth of monolayers of primary pancreatic islet cells. Islet cells derived from dispersed human or rat islets were seeded in minimum essential medium (MEM) containing 5% FBS and 2% B-27 neuronal medium onto established monolayers of rat neurons on glass coverslips. The seeded islet cells exhibited excellent cell adhesion and spreading (Fig. 1a), far surpassing the quality of islet cell monolayer cultures we had previously produced on HTB-9 or bovine corneal epithelial cell ECM^12^. Islet cells seeded in the neuronal culture medium on poly-ornithine coated glass but without neurons failed to adhere (Fig. 1b). The islet cells cultured with neurons formed an interconnected network with readily apparent neuronal-islet cell contacts observed by immunostaining for insulin, the beta cell and GABA-ergic neuronal cell marker GAD65, and the neuronal cell marker MAP2 (Fig. 1c,d). Using transmission electron microscopy (TEM), we identified neuronal axons forming synapse-like structural connections with islet endocrine cells, including insulin producing beta cells (Fig. 1e). The islet-neuron co-culture experiment, in addition to raising intriguing questions for future studies of the effects of their connectivity, established that factors in the co-culture system enabled primary islet cells to adhere and form stable islet cell monolayers on glass substrates.

### In the absence of neurons, defined surface coating and neuronal culture medium promote monolayers of well-spread and adherent islet cells on glass

Based on the adhesion and spreading of dispersed islet cells on glass surfaces in the presence of primary neurons and neuronal culture medium, we hypothesized that in the absence of neurons, surface-coating with purified well defined extracellular matrix molecules combined with neuronal culture medium might enable the adhesion of primary islet cells on glass and preserve primary islet cell phenotype and beta cell function to allow for detailed observation by super-resolution light microscopy. Integrin subunit β1 is highly expressed in islet cells^2^,^11^,^33^,^34^,^35^,^36^ and mediates islet cell-adhesion to laminin^37^ and collagen IV^34^, which are abundant in the ECM of native islets^38^. We investigated the potential of purified laminin or collagen IV to replace the co-culture with neurons in the presence of normal islet culture medium or culture medium formulated for primary neurons. Further experiments were performed to investigate beta cell function and validate the method for high-resolution microscopy.

**Figure 2:**
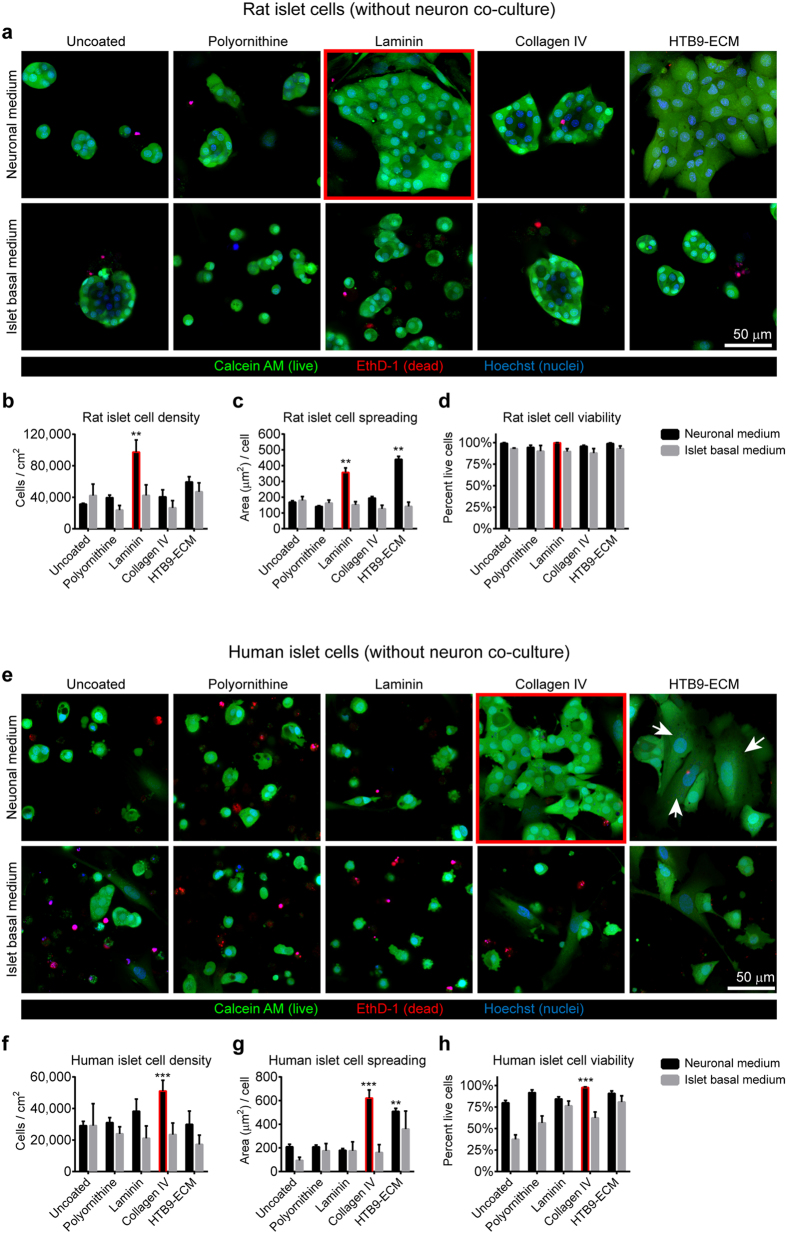
Optimization of primary islet cell monolayer culture conditions in the absence of neurons. Monolayer cultures of rat (**a**–**d**) and human (**e**–**h**) islet cells 4 days after seeding onto glass coverslips with different coatings without neuronal co-culture. (**a**,**e**) Representative confocal images of rat (**a**) and human (**e**) islet cells grown in neuronal media stained with calcein AM (live cells), ethidium homodimer 1 (dead cell nuclei) and counterstained by Hoechst 33342 (total nuclei). White arrows indicate fibroblast-like non endocrine cells. (**b**–**d**,**f**–**h**) Quantification of islet cell density (**b**,**f**), islet cell spreading (**c**,**g**) and islet cell viability (**d**,**h**) for rat (**b**–**d**) and human (**f**–**h**) islet cells cultured in neuronal or islet basal media. Mean ± SEM (*n* = 6 image random fields per condition). Statistical analysis by one-way ANOVA (**p < 0.01, ***p < 0.001, Tukey’s post-hoc pairwise comparisons of coated versus uncoated cover slips in the same medium condition).

Glass coverslips were first coated with laminin or collagen IV, and then seeded with dispersed neonatal rat or adult human islets cells in either neuronal medium or islet basal medium. We also prepared and similarly seeded uncoated coverslips, and coverslips coated with polyornithine or HTB-9 matrix. Both human and rat islets cells required culture in neuronal medium for strong attachment and spreading of constituent islet cells, but failed to adhere and spread well to any glass surface coating when cultured in islet basal medium (Fig. 2). When grown in neuronal medium, rat islet cells attached and spread well on both laminin and HTB-9 matrix but not on other surface coatings (Fig. 2a–d). Rat islet cell density was 40% higher on laminin than on HTB-9 matrix (Fig. 2b). Laminin and HTB-9 matrix supported comparable levels of rat islet cell spreading (Fig. 2c). Viability of rat islet cells was high across all conditions (Fig. 2c). In contrast to rat islet cells, human islet cells grown in neuronal medium attached and spread best on collagen IV and did not adhere well to laminin (Fig. 2e–h). Human islet cell spreading on HTB-9 matrix was higher than for the other conditions except for collagen IV, but human islet cell density on the HTB-9 matrix was low (Fig. 2f,g). Neuronal medium promoted increased viability of human islet cells compared to islet basal medium on all surface coatings (Fig. 2h). The HTB-9 matrix additionally promoted the attachment and proliferation of contaminating fibroblast-like cells, particularly from human islets. These fibroblast-like cells were also observed in low numbers in human islet cells cultured on collagen IV, but did not appear to interfere with endocrine cell attachment or survival. For long-term culture of human islet cell monolayers, we observed the ability of the chemotherapeutic drug ARA-C to rapidly eliminate fibroblast-like cells in islet cells cultures (Supplementary Fig. S1). However, we rarely observed fibroblasts in long term islet cell cultures and did not include ARA-C in further experiments.

### Monolayer primary islet cell cultures retain key characteristics of differentiated endocrine cells

**Figure 3:**
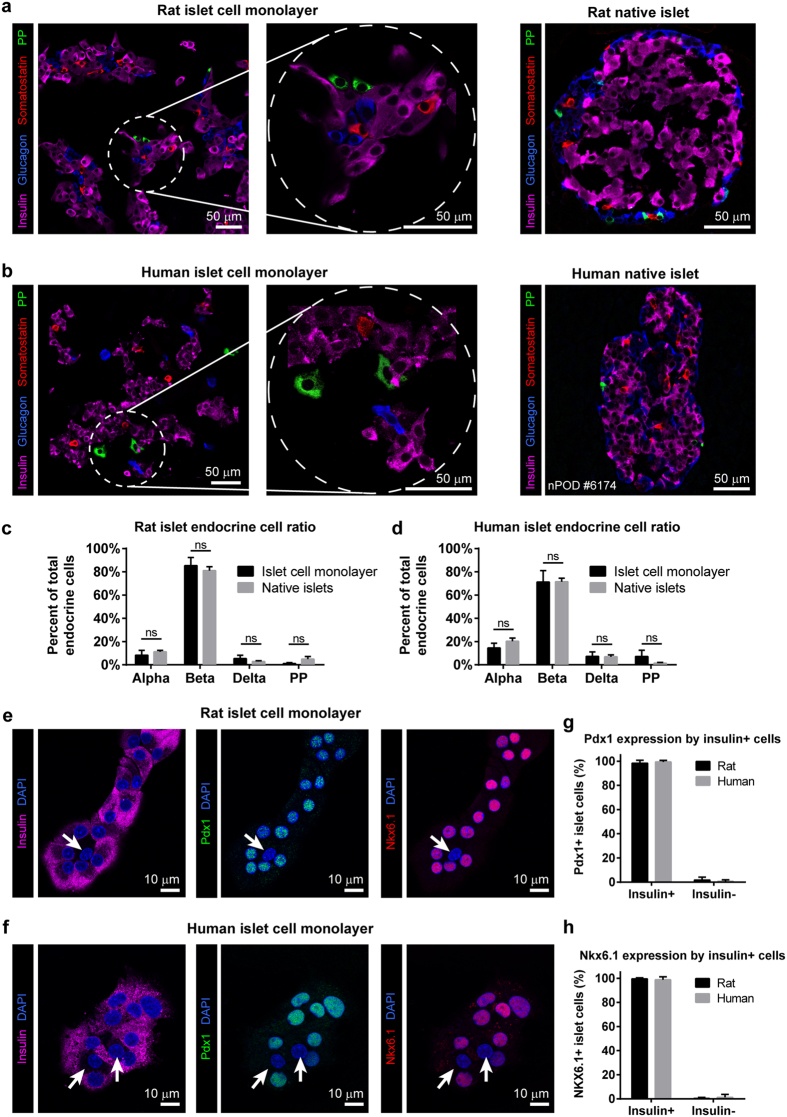
Maintenance of endocrine cell phenotype in primary islet monolayer cultures. (**a**) Rat islet cells cultured for four days on laminin-coated coverslips in neuronal medium and (**b**) human islet cells cultured for four days on collagen IV-coated coverslips in neuronal medium and immunostained for glucagon, insulin, somatostatin and pancreatic polypeptide (PP) to identify islet alpha, beta, delta and PP cells, respectively. Left image panels show monolayer islet cell cultures. Middle panels show increased magnification of the encircled regions in the left panel. Right panels show native islets from sections of rat and human pancreas. (**c**,**d**) Quantification of cell type distribution in rat (**c**) and human (**d**) islet cell monolayers and pancreatic tissue sections. Mean ± SEM (*n* = 6 random image fields from each of 2 different donors). Statistical analysis by Student’s *t*-test (ns, no significant difference). (**e**,**f**) Expression of beta cell markers PDX1 and NKX6-1 persists 2 weeks after seeding in monolayers in rat (**e**) and human (**f**) cultures. Arrows indicate lack of PDX1 and NKX6.1 expression in insulin-negative cells. Mean ± SEM (*n* = 6 random image fields). Images are representative of 3 independent experiments. (**g**) Quantification of PDX1 and (**h**) NKX6.1 expression in insulin-positive and insulin-negative islet cells in rat and human islet cell monolayer cultures.

Human and rat islet cell monolayers organized themselves into clusters of differentiated endocrine cells expressing the hormones glucagon, insulin, somatostatin and pancreatic polypeptide in alpha, beta, delta, and PP cells, respectively, in proportions similar to native islets^39^,^40^ (Fig. 3a–d). Insulin-positive beta cells in human and rat islet cell monolayers maintained high nuclear expression of the beta cell markers Pdx-1 and NKX-6.1 after 2 weeks of monolayer culture (Fig. 3e–h). No bi-hormonal expressing cells were observed. After a week of culture, considerable proliferation of neonatal rat islet beta cells grown on laminin was observed in neuronal but not in islet basal medium by Ki67 staining (Supplementary Fig. S2). In contrast, proliferation of adult human islet beta cells cultured on collagen IV coated glass in neuronal medium was extremely rare (Supplementary Fig. S2). Standard confocal microscopy of islet cell monolayers on glass immunostained for insulin, the neuroendocrine GABA-synthesizing enzyme GAD65 and the endoplasmic reticulum marker calnexin enabled detailed imaging of subcellular structures unresolvable in whole islets (Supplementary Fig. S2), and revealed beta cells adopting typical polarized polyhedron morphology^41^ and forming rosette-like micro-societies^41^ (Supplementary Fig. S2).

**Figure 4:**
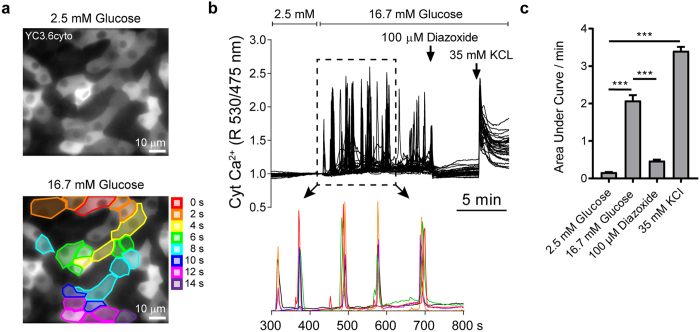
Functional calcium-responsive glucose sensing in islet monolayer cultures. (**a**) Rat islet cell monolayers cultured in neuronal medium and transduced with adenovirus to express Cameleon cytosolic Ca^2+^ sensor YC3.6_cyto_ under the control of the rat insulin promoter. Top panel shows localization of YC3.6_cyto_ in the cytosol. Bottom panel shows wavelike propagation of Ca^2+^ signaling between adjacent beta cells during high glucose stimulation. See also Supplementary Video 1. (**b**) A representative Ca^2+^ imaging experiment in primary rat beta cells showing simultaneously-recorded Ca^2+^ traces from 41 beta cells in a single field-of-view. The lower panel shows enlargement of a portion of the graph revealing strongly coordinated Ca^2+^ activity in subsets of beta cells during 16.7 mM glucose stimulation. Each color represents an individual cell recording (6 cell traces shown). Colors do not correspond to those in (**a**). (**c**) Quantification of the Ca^2+^ signaling per min during each phase of the experiments shown in (**b**). Mean ± SD (*n* = 3 independent experiments, 41–55 cells analyzed per experiment). Statistical analysis by two-way ANOVA (***p < 0.001, Tukey’s post-hoc pairwise comparisons).

To test the functionality of primary beta cells in islet cell monolayers, we acquired time-lapse videos of calcium signaling in primary rat beta cells expressing the cytosolic Cameleon Ca^2+^ sensor YC3.6cyto^42^ under the control of the rat insulin promoter (Ad-RIP-YC3.6cyto) (Fig. 4a–c). A wavelike propagation of calcium signals across neighboring cells was observed consistent with establishment and maintenance of cell-cell contacts and interactions in the monolayer cultures (Fig. 4a). The beta cells displayed characteristic islet-like calcium signaling behavior: calcium transients were absent under low glucose (2.5 mM) conditions, whereas rhythmic coordinated calcium spikes (Supplementary Video S1) were observed in response to glucose stimulation (16.7 mM). The high-glucose stimulated calcium signaling was abolished by maintaining the ATP-dependent K + channels in an open state using diazoxide. Membrane depolarization with KCl caused a large calcium rise (Fig. 4b). We compared calcium signaling in the beta cell population of islet cell monolayers on glass cultured in either islet basal medium or neuronal medium (Supplementary Fig. S3, Supplementary Videos S2, S3). Islet cell monolayers cultured in neuronal medium involved larger colonies of well extended interconnected cells than cultures in islet basal medium where cells were round and less well adhered. While calcium signaling in response to high glucose stimulation was similar in magnitude in the two culture media, only beta cells grown in neuronal medium exhibited a complete recovery of basal calcium levels in-between calcium transients (Supplementary Fig. S3). Together, the data indicate that established characteristics of beta cell signaling in response to glucose are well maintained in our monolayer culture system on glass in neuronal medium.

### STED and SIM imaging of primary islet cell monolayer cultures on glass reveals superior resolution of insulin granules and microtubule and actin networks

**Figure 5:**
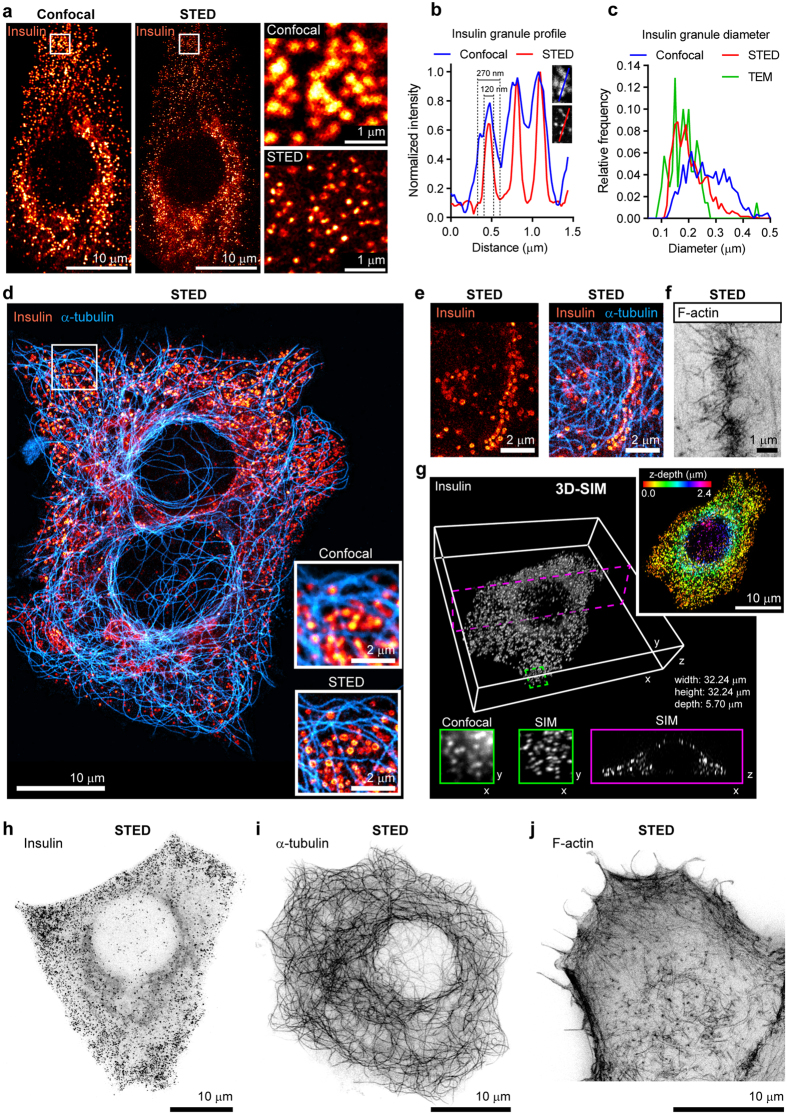
STED and SIM microscopy techniques applied to islet cell monolayers on glass reveal superior resolution of insulin granules and the microskeleton. (**a**) Representative confocal and STED super-resolution microscopy images of rat islet cell monolayers immunostained for insulin. Right panels show increased magnification of the boxed regions in the left panels. (**b**) Representative intensity profile measurement across a group of three insulin granules according to confocal or STED microscopy. Feature sizes measured as full width at half maximum. (**c**) Size distribution of insulin granules as measured by confocal, STED or TEM microscopy. (**d**) Comparison of confocal and STED super-resolution microscopy portrait of a human islet beta cell monolayers immunostained for insulin and α-tubulin. Inset frames show higher magnification of boxed region imaged by either confocal or STED microscopy. (**e**) STED image of insulin granule recruitment and docking to plasma membranes at a cell-cell interface in rat islet cell monolayers following a brief exposure to high glucose. (**f**) STED image of the cortical F-actin network at a cell-cell interface in human islet cell monolayers. (**g**) 3D-SIM super resolution microscopy of a human islet beta cell, immunostained for insulin. Central black and white panel shows a super-resolution three-dimensional imaging of a SIM z-stack acquisition of insulin staining in a single human beta cell. Bottom left two inset frames show the same single x-y plane with higher magnification of the green boxed region, imaged by either confocal or SIM microscopy. Bottom right inset frame shows a single x-z plane of the magenta boxed region imaged by SIM microscopy. Top right frame shows the same cell imaged by SIM microscopy with insulin immunostaining color-coded for depth in the z-axial direction. (**h**) Representative STED image of insulin granules in a human beta cell. (**i**) A representative STED image of α-tubulin in a human beta cell. (**j**) A representative STED image of F-actin in a rat beta cell.

We next performed STED super-resolution imaging of insulin granules, microtubules and actin filaments in primary rat and human beta cells (Fig. 5 and Supplementary Fig. S4). The size of insulin granules and cytoskeletal features imaged by STED demonstrate successful sub-diffraction limit imaging of cellular structures below the resolving power of traditional light microscopy (Fig. 2a–c), in agreement with measurements made by transmission electron microscopy (TEM) (Supplementary Fig. S5). STED imaging of insulin in human beta cells that were fixed following a brief incubation in high glucose revealed individual insulin granules docked at the plasma membrane (Fig. 5e). We observed that the cell-cell interface between adjacent beta cells is characterized by a dense cortical actin network (Fig. 5f), which is implicated in the recruitment and docking of secretory granules^41^. We also captured three-dimensional (3D) super-resolution images of human islet beta cells immunostained for insulin using the alternative super-resolution method of structured illumination microscopy (SIM)^43^ (Fig. 5g). The resolving power of both STED and SIM super-resolution microscopy techniques when performed on primary beta cells on glass enabled clear distinction of individual insulin vesicles. When viewed by traditional confocal microscopy, neighboring insulin granules often appear as merged blobs and larger in size than the real structure. For sub-cellular structures as transport vesicles and cytoskeletal components that are smaller than the resolving length scale of traditional confocal microscopy, super-resolution optical microscopy provides a more accurate and detailed visualization of the specimen.

### Evaluation of primary cilia in the context of enhanced primary beta cell proliferation

**Figure 6:**
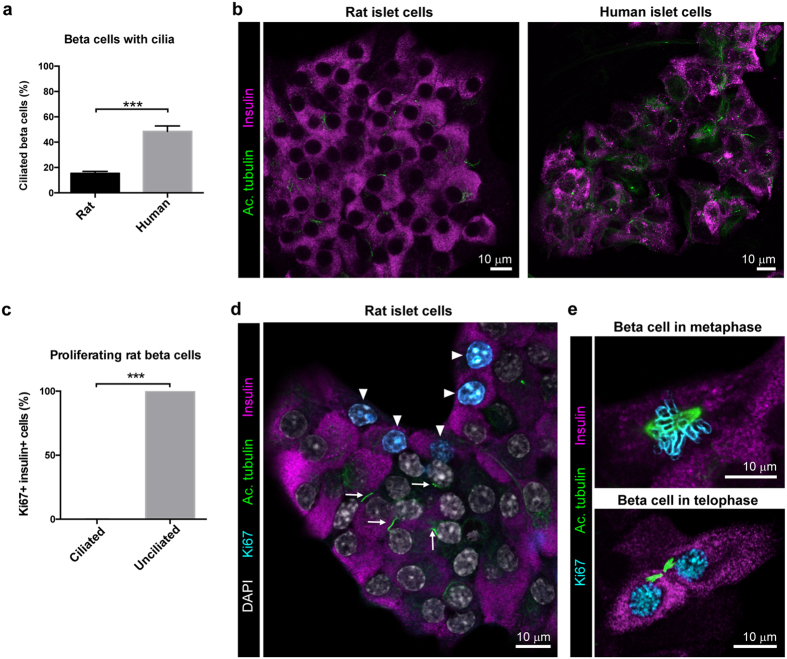
Presence of primary cilia inversely correlates with beta cell proliferation. (**a**) Percentage of insulin-positive beta cells with primary cilia in rat and human islet monolayer cultures. Mean ± SEM (*n* = 10 random image fields from each of two different donors). Statistical analysis by Student’s *t*-test (***p < 0.001). (**b**) Representative confocal images of rat and human islet cells immunostained for insulin and the cilia marker acetylated alpha tubulin. (**c**) Percentage of proliferating beta cells positive for both Ki67 and insulin with and without primary cilia in rat islet cells. Mean ± SEM (*n* = 40 random image fields with ~50 cells per field). Statistical analysis by Student’s *t*-test (***p < 0.001). (**d**) A representative confocal image showing unciliated proliferating Ki67-positive rat insulin-positive cells (arrowheads) and a group of Ki67-negative beta cells with cilia (arrows). (**e**) Confocal images showing the localization of acetylated tubulin during metaphase and telophase in mitotic beta cells.

As noted above, primary cilia have been implicated as proliferation suppressors that help maintain beta cell quiescence and homeostatic functionality. Taking advantage of the technical developments described above, and illustrating its utility, islet cell monolayer cultures on glass were used to observe primary cilia by immunostaining for the cilia markers acetylated alpha tubulin and pericentrin, which mark the cilium-originating point at the centrosome (Supplementary Fig. S6 and Fig. 6a,b). The new methodology enabled identification of individual cilia-expressing beta cells and analyses of the lengths and orientation of their cilia (Supplementary Fig. S6).

We noted that the percentage of ciliated beta cells was 3 fold higher in human islet cell monolayers compared to neonatal rat islet cell monolayers (48% vs 16% of insulin-positive cells) (Fig. 6a,b). Because analyses of expression of the proliferative marker Ki67 indicated a lower rate of proliferation of adult human beta cells than neonatal rat beta cells (Supplementary Fig. S2), we speculated that the lower incidence of primary cilia in rat beta cells might reflect differences in cell cycle status between the two cultures. In support of this hypothesis, we observed that primary cilia were uniformly absent from proliferating Ki67-positive rat islet beta cells (Fig. 6c,d). Furthermore, images of primary beta cells undergoing cell division showed acetylated alpha tubulin localized to the mitotic spindle during mitosis rather than in ciliary structures (Fig. 6e). Together these observations suggest that the presence of primary cilia is incompatible with cell cycle progression in primary beta cells.

**Figure 7:**
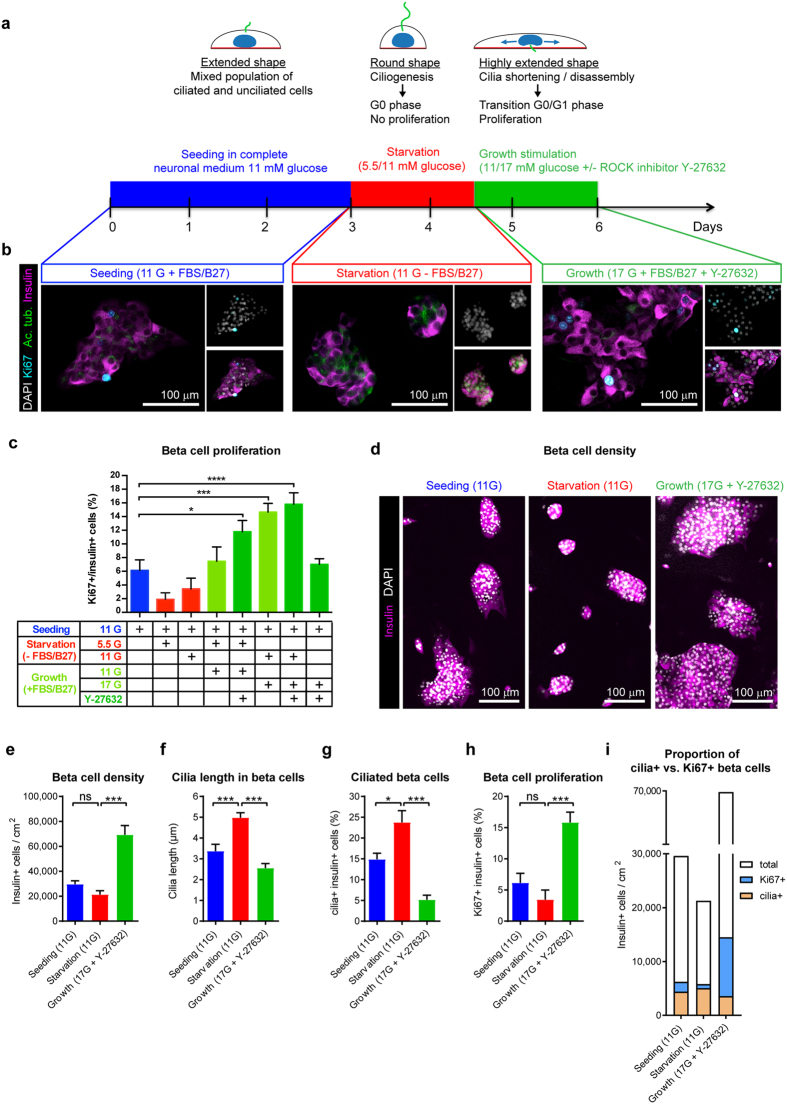
Cilia expression in the context of enhanced beta cell proliferation. (**a**) Schematic representation of a protocol based on nutrient withdrawal (starving) and re-introduction (growth stimulation) to affect proliferation and cilia expression in beta cells. (**b**) Representative fluorescence images of rat islet monolayer cells at different stages of the protocol described in (**a**) and immunostained for DAPI, Ki67, acetylated tubulin and insulin. (**c**) Beta cell proliferation during different stages and permutations of the protocol. Mean ± SEM (*n* = 10 random image fields per group). Statistical analysis by one-way ANOVA (*p < 0.05, ***p < 0.001, ****p < 0.0001, Dunnett’s pairwise comparisons relative to Seeding condition). (**d**) Representative fluorescence images of rat islet monolayer cells in three different stages of the most effective growth stimulatory protocol, immunostained for DAPI and insulin. (**e**–**h**) Quantification of (**e**) beta cell density, (**f**) cilia length, (**g**) percentage of ciliated beta cells and (**h**) percentage of beta cell proliferation and (**i**) visualization of proportions of cilia + cells and Ki67 + cells respectively in beta cells in the three stages. Mean ± SEM (*n* = 10–20 random image fields per group). Statistical analysis by one-way ANOVA (*p < 0.05, **p < 0.01, ***p < 0.001, ns no statistical difference, Tukey’s post-hoc pairwise comparisons). The three groups of cells in d-i were cultured for a total of 144 hours before counting of cells with media change in each at 72 and 108 hours. In the first group, cells were cultured in neuronal medium with 11 mM glucose for 144 hours. In the second group, cells were cultured in neuronal medium with 11 mM glucose for 108 hours and in the same medium without serum and B-27 for 36 hours. In the third group, cells were cultured in neuronal medium with 11 mM glucose for 72 hours, in the same medium without serum and B-27 for 36 hours and in neuronal medium with 17 mM glucose and ROCK inhibitor for 36 hours. Analyses were performed in triplicate. Data are representative of two independent experiments.

We next asked if adaptation of a two-step protocol previously shown to drive cilia disassembly based on nutrient withdrawal and reintroduction in non-islet cells^44^ could be exploited as a mechanism to induce proliferation of primary beta cells (Fig. 7a). Low glucose concentration causes beta cells to become more rounded, while high glucose culture medium causes the opposite effect, enhancing the adhesion and spreading of primary beta cells^45^. We first tested the effect of high and low glucose on cell shape in our rat islet cell monolayer cultures and confirmed a marked effect on islet cell shape resulting from switching the glucose concentration from 11 mM to 5.5 mM (rounded cells) and then from 5.5 mM to 11 mM (extended well-adhered cells) (Supplementary Fig. S7 and results not shown). Cytoskeletal rearrangements, occurring when cells transition from a rounded to a highly-spread shape, have been shown to drive cilia reabsorption in human retinal cells and promote re-entry into the cell cycle^46^. Furthermore, the ROCK inhibitor Y-27632, which induces a repositioning of the basal bodies below the nucleus, thus reducing ciliogenesis^46^, has been reported to have mitogenic effects on beta cells^24^. We focused these experiments on neonatal rat islet cell monolayer cultures due to the rarity of Ki67-positive beta cells in human islet monolayer cultures.

Neonatal rat islet cell monolayers were established in neuronal medium containing 11 mM glucose. After three days in culture, cells were subjected to serum and B-27 starvation for 36 hours to induce beta cell rounding and cilia growth in either 5.5 mM or 11 mM glucose. Then, serum and B-27 were reintroduced and glucose concentration increased from 5.5 mM to 11 mM or from 11 mM to 17 mM in the presence or absence of the ROCK inhibitor Y-27632 (Fig. 7a,b). Fluorescence analyses of cells at the different stages of the protocol revealed a rounding effect of serum and B-27 starvation in 11 mM glucose, which was reversed during the growth stimulation phase (Fig. 7b). Analyses of Ki67 expression at different stages and permutations of the protocol (Fig. 7c), revealed ~6% of beta cells proliferating in the initial seeding phase. The percentage of proliferating cells decreased during the starvation phase but rebound during the growth stimulatory phase. Thus, increasing glucose from 11 mM to 17 mM following the starvation phase significantly increased the percentage of beta cell proliferation over the initial seeding with ~15% of beta cells being Ki67 positive. Addition of the ROCK inhibitor Y-27632 enhanced the proliferative effect slightly but not significantly to ~16% in this condition. Increasing the glucose concentration to only 11 mM in the growth stimulation phase resulted in a non-significant increase over the seeding phase in the percentage of proliferating cells. In this condition however, combining an increase in glucose concentration with addition of Y-27632 resulted in a significant increase in proliferation over the seeding phase, with ~12% of beta cells being Ki67 positive (Fig. 7c), suggesting that inhibition of ROCK may exert an additional influence on proliferation. Omitting the starvation step obviated the increased percentage of proliferation seen in the growth stimulation phase.

We selected for further evaluations the most effective proliferation protocol, involving 17 mM glucose plus the ROCK inhibitor following serum plus B-27 starvation in 11 mM glucose. Cell density measurements of monolayer cultures (Fig. 7d,e) revealed the ability of this protocol to increase beta cell density. Initial seeding density was ~30,000 cells/cm^2^, which decreased to ~20,000 cell/cm^2^ during starvation in 11 mM glucose. In the growth-stimulatory phase, beta cell mass was significantly increased, to ~70,000 cells/cm^2^. We asked whether the effect on proliferation was associated with changes in cilia expression and/or length (Fig. 7f–i). Following initial seeding, primary cilia were expressed in ~15% of beta cells (~4500 cells/cm^2^), and the average cilia length was ~3 μm. At the end of starvation in 11 mM glucose, the percentage of cilia expressing cells had increased to ~24% of remaining beta cells (4800 cells/cm^2^) and average cilia length had increased to ~5 μm. Following re-introduction of serum plus B-27, increasing glucose to 17 mM, and adding the ROCK inhibitor for 36 hours, cilia expression decreased to ~5% of beta cells (~3500 cells/cm^2^) and the cilia length decreased to ~3 μm (Fig. 7f,g,i). Thus, loss of cilia expressing cells amounted to ~1300 cells/cm^2^ during the growth stimulatory phase. In comparison, during the starvation phase, the percentage of proliferating cells (Ki67 positive cells) decreased from 6% (~1800 cells/cm^2^) to 3.5% (~750 cells/cm^2^) and then increased to 16% (~11,000 cells/cm^2^) (Fig. 7h,i) during the growth stimulatory phase further substantiating the benefits of the protocol for increasing the proliferating pool of beta cells. Thus, loss of cilia expressing beta cells amounted to ~1300 cells/cm^2^ during the growth stimulatory phase while the increase in the number of proliferating beta cells amounted to ~10,000 cells/cm^2^.

This increased number of proliferating Ki67 positive cells during the growth stimulatory phase (Fig. 7i) likely reflects contributions by three distinct subsets of beta cells, including descendants of cells that (i) lost cilia during the growth-stimulatory phase; (ii) were already cilia negative but growth arrested (by the lack of mitogenic factors) until re-addition of nutrients and mitogens or (iii) were already cilia-negative, and Ki67-positive at the end of the starvation period. In summary, while starvation caused cilia to grow in length, re-addition of serum/B27, increasing glucose and adding ROCK inhibitor resulted in a burst in beta cell proliferation that was associated with reductions in cilia length and the number of cilia-expressing cells, consistent with a growth suppressive role.

## Discussion

We have developed a simple and easily reproducible method for two-dimensional culture of primary human and rat islet cells on glass. Critically, the combination of neuronal culture medium containing B-27 neuronal growth supplement together with a collagen IV (human islet cells) or laminin (rat islet cells) surface coating elicits the formation of well-spread and robustly-attached primary islet cell monolayers. The B-27 supplement contains a cocktail of growth factors and antioxidants formulated to support the viability of central nervous system neurons. Several B-27 components, including retinoic acid^47^, insulin^48^ and triiodothyronine^49^, are known to support islet cell function and survival, while superoxide dismutase, glutathione and corticosterone may reduce cellular stress resulting from the dispersion of whole islet into single cells.

Pancreatic islet cells share many features with neurons, including the secretion of neurotransmitters and expression of neurotransmitter receptors for auto and paracrine coordination of islet function^50^,^51^. The commonly used islet culture medium RPMI 1640 contains high concentrations of neuroactive amino-acids, L-Glutamate, L-Aspartate, and L-Cysteine. These amino acid components result in RPMI medium having an excitatory neurotoxic effect, with consequent poor viability of primary neurons^52^. As islet cells also express neurotransmitter receptors^51^, the amino acid content of RPMI may induce similar toxic effects on dispersed islet cells. The use of a neuron-compatible culture medium such as Neurobasal Medium or MEM, which are intentionally formulated without neuroactive amino acids^53^, however, resulted in highly viable islet cell cultures.

Cell-ECM^35^ and cell-cell^19^,^54^ interactions are critical for islet viability and function. In the new culture system, cell-ECM and cell-cell interactions are partially restored through defined matrix coatings, which ligate beta cell integrin receptors, and encourage the propensity of dissociated islet cells to coalesce into two dimensional micro-societies of clustered endocrine cells. We found that collagen IV surface coating preferentially supports human islet cell adhesion and formation of micro-societies, whereas laminin and to a lesser extent the HTB-9 surface coating preferentially support rat islet cell adhesion and assembly in two dimensional clusters. The lack of adhesion of human islet cells on laminin and HTB-9 is consistent with laminin being a major constituent protein in HTB-9 extracellular matrix. The species-specific preferential adhesion to collagen IV or laminin is likely a reflection of differential expression of alpha-beta integrin pairings in human and rat islets^35^ and may imply the existence of underlying species differences in islet cell-matrix architecture.

When whole islets are dispersed into individual cells, connections are lost and the cells often display reduced or dysregulated calcium signaling and insulin-secretory capability^55^. The analysis of calcium signals in our monolayer culture system indicates that the beta cells have maintained full responsiveness to glucose. Furthermore, the wavelike propagation of calcium signals across neighboring beta cells within the islet cell monolayers is consistent with the proper establishment and maintenance of cell-cell contacts and interactions that are essential for nutrient activation of beta cells^19^. Strikingly, the cells analyzed showed no calcium signals under resting conditions. The beta cell activation that immediately followed initiation of the glucose stimulus was dependent on the metabolic triggering pathway of insulin secretion, as it was suppressed by the ATP-dependent K+ channel activator diazoxide. The calcium data provide strong evidence for the functional robustness of beta cells in this new monolayer culture system.

The new method described herein for culturing islet cell monolayers on glass has facilitated the visualization of primary cilia expression in beta cells by traditional confocal microscopy and revealed a correlation between lack of cilia expression in and proliferation of neonatal rat beta cells, suggesting that the inverse relationship described previously for other cell types may apply to beta cells. We identified a 2-step growth-stimulatory protocol that markedly increased proliferation of primary beta cells, involving factors that have been previously shown to reduce cilia in non-beta cells^44^. This result is interesting, because primary beta cells are characteristically refractory to proliferation in culture. In other cell types, it has been shown that cilia-regulated signaling pathways are inversely correlated with the cell cycle. Thus, in mouse embryonic fibroblasts, the presence of primary cilia restrains beta-catenin and Wnt cell-cycle signaling^56^, while ciliary disassembly leads to nuclear translocation of beta-catenin and activation of transcription factors responsible for cell cycle progression. In a different signaling pathway, inhibition of ROCK signaling can lead to cilia resorption through promoting cytoskeletal rearrangements, basal body repositioning and centrosome splitting^46^,^57^. Aurora Kinase A, which is mitogenic in beta cells^25^, also stimulates cell cycle progression by mediating cilia disassembly in other cell types^23^,^58^. Thus, there is strong evidence for inter-regulation between primary cilia and cell cycle progression. While the current study provides suggestive evidence that cilia expression and cell cycle progression may also be inversely correlated in beta cells, it has not formally shown that the beta cells that loose cilia are the same cells that go on to proliferate, although it seems likely that they contribute to the observed increase in cell number in the 2-step proliferation-stimulating regimen. Future development of lineage tracing tools for cilia expression in beta cells could enable such analyses.

The 2-step starvation/growth-stimulatory protocol that increased beta cell proliferation, was performed by first inducing cell rounding through a starvation step followed by markedly increasing cell spreading by re-addition of serum/B27 and increasing glucose. Cyclically applying this method to round and spread beta cells on appropriate surfaces could provide a procedure for continuing beta cell expansion while preserving differentiated phenotype. It is possible that this proliferation pathway has not been previously identified in islets because beta cells must first be cultured on two-dimensional surfaces to encourage a high degree of cell spreading. Beta cells may be less susceptible to stimuli that increase spreading and cilia resorption in the confined three-dimensional environment of intact islets, as compared to a two-dimensional monolayer culture with appropriate matrix and soluble factors, as exemplified by the system we have established.

The advancement in the two dimensional monolayer islet cell culture system described herein does not recapitulate the complete three dimensional environment of whole islets. However, it enables the subcellular-resolution of physiological processes in functional primary islet endocrine cells using live-cell and super-resolution microscopy that require cells to be adherent on thin glass surfaces for optimal visualization. With these imaging capacities, islet biology studies performed in primary islet cells can now benefit from the full array of possibilities offered by the latest advancements in light microscopy, particularly the visualization of molecular interactions and biochemical events at the sub-organelle level.

## Methods

### Islet isolation and culture

Rat islets were isolated from pancreases of P5 Sprague Dawley rats (Charles River) as previously described^12^ except using Liberase TL (Roche) for pancreas digestion. Hand-picked islets were cultured at 37 °C in 10 cm non-adherent cell culture dishes (500 islets/dish) in islet basal medium: RPMI 1640 medium with GlutaMAX (Gibco), 11 mM glucose, 10% fetal bovine serum (FBS), 1% Penicillin/Streptomycin.

Human pancreatic islets were obtained through the European Consortium on Islet Transplantation, (ECIT), Islets for Basic Research Program. Human islets were cultured at 24 °C in 10 cm non-adherent cell culture dishes (500 islets/dish) in CMRL medium with 2% glutamine, 10% FBS, 10 mM HEPES and 1% Penicillin/Streptomycin. Human islets used in this study were obtained from three normal non-diabetic donors, two male and one female, ranging in age 18–59 years with a body mass index of 20–29 kg/m^2^.

### Co-culture of islet cells and neurons

Primary rat hippocampal neurons were prepared from P2-P3 Sprague Dawley rats, as described by Codazzi *et al*.^59^. Neurons were seeded on round poly-L-ornithine-coated glass or Thermanox coverslips (Nunc), at 100,000 cells per coverslip in a 24-well plate and in 1 ml of **neuronal medium**: Minimum essential medium (MEM) with GlutaMAX (Gibco), 11 mM glucose, 5% FBS, 1 mM sodium pyruvate, 10 mM HEPES and 1x B-27 Supplement^53^ (Gibco). One day after isolation, 3 μM of the chemotherapeutic agent ARA-C (Sigma-Aldrich) was added to the culture medium to eliminate astrocytes and obtain a neuronal culture of high purity (>90% neurons). Three-four days after preparation of a neuronal culture, without changing the medium, a suspension of either human or rat islet cells was prepared as described below, and 50,000 islet cells in 50 μL of neuronal medium were added per well to the established neurons.

### Preparation of matrix-coated coverslips

Lab-Tek 4-well Chambered Coverglass (Nunc, Thermo Fisher Scientific) were used for live/dead cell staining. Round 12 mm diameter and 0.17 mm thickness borosilicate glass coverslips (Electron Microscopy Sciences) were used for immunofluorescence staining. Coverglass-bottom Fluorodishes (World Precision Instruments) and MatTek dishes (MatTek Corporation) were used for live cell imaging experiments. Glass surfaces were coated with purified laminin (Gibco) or purified collagen IV (Sigma Aldrich) at 50 μg/ml in HBSS with Ca^2+^/Mg^2+^ for 1 hour at 37 °C, then washed 3x in HBSS with Ca^2+^/Mg^2+^. Glass coverslips were coated with Poly-L-ornithine at 400 μg/ml in pyrogen-free water for 1 hour at 37 °C then washed 3x with pyrogen-free water. HTB-9 matrix was generated from HTB-9 human bladder carcinoma cells (ATCC) grown to 95% confluence on glass coverslips, lysed with 20 mM NH_4_OH + 0.5% Triton X-100 in pyrogen-free water, followed by three washes in pyrogen-free water.

### Preparation of human and rat islet cell monolayer cultures without neurons

Within one week of isolation, rat or human islets were hand-picked from suspension cultures, collected in a 15 ml tube and washed twice in PBS without Ca^2+^ and Mg^2+^ (Gibco). Islets were dissociated into a suspension of single islet cells by continuous gentle pipetting in 0.3 ml 0.05% trypsin-EDTA (Gibco) per 500 islets for 3 minutes at 37 °C. Trypsin digestion was halted by adding neuronal or islet basal medium to a total volume of 15 ml, followed by pelleting of islet cells by centrifugation for 5 min at 1400 rpm (350 × g) and resuspension in neuronal or islet basal medium. Islet cells were seeded on matrix-coated surfaces at approximately 35,000 cells/cm^2^ in neuronal medium for all experiments except for groups indicated as seeded in islet basal medium. Islet cells required 3–4 days of culture to adhere and spread on surfaces before further experimentation. While typically not necessary, elimination of fibroblast like cells in human islet cell monolayer cultures maintained for 7 days or longer, could be achieved by adding 3 μM of ARA-C to the culture medium one day after islet cell seeding. ARA-C was used for establishing the neuronal cultures seeded with islet cells in Fig. 1 and for islet cell monolayer cultures shown in Supplementary Figure S1.

### Live/dead cell staining

Live/dead staining was performed using the LIVE/DEAD^®^ cell viability kit for mammalian cells (Molecular probes) with addition of Hoechst 33342 (Molecular Probes). Live cells were distinguished by the enzymatic conversion of non-fluorescent cell-permeant calcein acetoxymethyl (AM) ester to fluorescent calcium within live cells. Dead cells were distinguished by the entry of cell-impermeant ethidium homodimer-1 into cells with compromised membranes where it increases fluorescence 40-fold upon binding nucleic acids. Total cells were distinguished by the addition of cell-permeant Hoechst 33342, a nuclear counterstain that fluoresces blue when bound to double-stranded DNA. Calcein AM (2 μM), ethidium homodimer-1 (4 μM) and Hoechst 33342 (8 μM) were added directly to cell monolayers without removing culture media to avoid removal of non-adherent cells. Cells were incubated for 30 minutes at room temperature and imaged live with a Zeiss LSM700 confocal microscope with 20x/0.8 NA Plan-Apochromat air objective. Analyses of cell adhesion, spreading and viability were performed in ImageJ^60^.

### Immunofluorescence staining

Human pancreas sections obtained from the Network for Pancreatic Organ Donors with Diabetes (nPOD case ID #6174 and 6230) were deparaffinized followed by acidic-pH heat-mediated antigen retrieval according to the nPOD standard operating procedure for immunopathology^61^. Whole islets, monolayers of pancreatic islet cells and co-cultures of islet cells and neurons were fixed with 4% EM-grade PFA (Electron Microscopy Sciences) at room temperature for 20 minutes or 1 hour, respectively. Samples were blocked and permeabilized in PBS + 0.3% Triton X-100 with 10% goat or donkey serum. Primary antibodies (listed below) were incubated overnight at 4 °C. Alexa Fluor 405, 488, 568, and 647 conjugated secondary antibodies (Molecular Probes) were incubated at 1:200 dilution in 0.3% Triton X-100 in PBS for 30 minutes at room temperature. Coverslips were mounted with ProLong Gold Antifade Reagent (Molecular Probes). Immunostained whole islets were handled in 1.5 ml Eppendorf tubes by centrifugation between immunostaining steps and mounted beneath glass coverslips.

For alpha-tubulin staining, cells were washed in 37 °C PHEM buffer (60 mM PIPES, 25 mM HEPES, 10 mM EGTA, 4 mM MgSO_4_), fixed for 10 min at 37 °C in 3.2% EM-grade PFA (Electron Microscopy Sciences) with 0.05% EM-grade glutaraldehyde (Electron Microscopy Sciences), washed three times with PBS and permeabilized for 15 min with 0.3% TX-100 in PBS. Unreacted aldehydes were quenched by 3 × 10 min washes with 1 mg/mL sodium borohydride in PBS. Immunostaining was performed as above.

### Antibodies

The following unconjugated primary antibodies were used for immunofluorescence staining: guinea pig anti-insulin (Linco 4011-01) 1:10,000 (for STED and SIM microscopy), chicken anti-insulin (Abcam ab14042) 1:2000 (for multi-color confocal microscopy), GAD6, a mouse monoclonal antibody specific for GAD65^62^ 1:1000, chicken anti-MAP2 (Abcam ab5392) 1:10,000, sheep anti-somatostatin (Abcam ab35425) 1:200, rabbit anti-pancreatic polypeptide (Abcam ab113694) 1:1000, rabbit anti-Ki67 (Abcam ab16667) 1:100, mouse anti-beta actin (Sigma A1978) 1:500, rabbit anti-calnexin (Abcam ab22595) 1:200, rabbit anti-alpha-tubulin (Abcam ab18251) 1:200, guinea pig anti-PDX1 (Abcam ab47308) 1:2000, mouse anti-NKX6-1 (Developmental Studies Hybridoma Bank F55A12s) 1:6, mouse anti-acetylated alpha tubulin (Sigma T7451.) 1:1000, rabbit anti-pericentrin (Abcam ab4448) 1:5000.

### Microscopy

Standard confocal microscopy was performed on multi-color immunostained samples on a Zeiss LSM700 confocal microscope with 20x/0.8 NA Plan-Apochromat air, 40x/1.30 and 63x/1.40 NA Plan-Apochromat oil-immersion objectives.

Super-resolution microscopy was performed on rat and human islet cell monolayers cultured on laminin (for rat) or collagen IV (for human) coated 0.17 mm thickness glass coverslips. Cells were immunostained for insulin detected with Alexa Fluor 488 secondary antibody and alpha tubulin detected with Alexa Fluor 532 secondary antibody; or stained for actin with Alexa Fluor 488 phalloidin (Molecular Probes).

STED super-resolution microscopy was performed on Leica TCS SP5 STED CW white light laser (WLL) and Leica SP8 STED 3X microscopes with HC PL APO 100x/1.40 NA oil immersion objectives, 488 nm WLL excitation line with 592 nm depletion laser or 532 nm WLL excitation line with 660 nm depletion laser, Hybrid detector (HyD) with timing gate 1.0–6.5 ns, and with 21 nm/pixel xy resolution. Images were processed and measured in ImageJ^60^.

3D-SIM microscopy was performed on a Nikon N-SIM configured Nikon Eclipse Ti inverted microscope with Apochromat total internal reflection fluorescence (TIRF) 100x/1.49 NA objective and electron-multiplying charge-coupled device camera (EMCCD, IXON3; Andor Technology) at 512 × 512 pixels with 63 nm/pixel resolution in the x,y coordinates and 60 nm/voxel resolution in the z coordinate. Super-resolution images were reconstructed using built-in algorithms of NIS-Elements software^63^. Reconstruction parameters were: contrast 0.70; apodization 1.00; and Widh3DFilter 0.20.

Cytosolic Ca^2+^ was measured in monolayer cultures of rat islet cells transduced with adenovirus encoding the Cameleon ratiometric Ca^2+^ sensor YC3.6_cyto_^42^ under the control of the rat insulin promoter (Ad-RIP-YC3.6_cyto_)^20^. Rat islet cells, cultured for four days in either neuronal or islet basal medium on laminin-coated MatTek dishes, were infected with 3 × 10^6^ infectious units (IFU) Ad-RIP-YC3.6_cyto_ adenovirus for 24 h at 37 °C. Two days after infection, rat islet cell monolayers were washed 4 times in Krebs-Ringer bicarbonate HEPES buffer (KRBH): 140 mM NaCl, 3.6 mM KCl, 0.5 mM NaH_2_PO_4_, 0.5 mM MgSO_4_, 1.5 mM CaCl_2_, 10 mM HEPES, 5 mM NaHCO_3_, 2.5 mM glucose, pH 7.4. Calcium imaging was performed in a thermostatic chamber at 37 °C on a DMI6000 B inverted fluorescence microscope with HCX PL APO 40x/1.30 NA oil immersion objective (Leica), Evolve 512 EMCCD (Photometrics), BP436/20 nm excitation filter, and BP480/40 nm and BP535/30 nm emission filters. Small volumes of concentrated solutions were added by hand to cells in KRBH at the given time points to produce a final concentration of 16.7 mM glucose, 100 μM diazoxide, or 35 mM KCl. Fluorescence ratios were calculated in MetaFluor 7.0 (Meta Imaging Series). In order to compensate for an uneven baseline, we used a background estimation by minimizing an asymmetric truncated quadratic 3rd order polynomial function provided by Vincent Mazet on Matlab Central^64^,^65^. The Area under curve was then calculated by summing the difference between the original signal and the estimated background point by point along the two curves. The resulting number was then divided by the length of the acquisition in minutes to obtain the reported measurement of area under curve per min.

Counting of primary cilia in beta cells and analyses of cilia length were performed on images acquired with a Leica DM5500B fluorescence microscope with a 40x air objective. Analyses of beta cell density were performed on images captured with Olympus Slide Scanner VS120-L100 with a 10x/0.40 UPLSAPO air objective.

For transmission electron microscopy (TEM), co-cultures of islet cells with neurons or whole rat islets were fixed in 2.5% glutaraldehyde/2.0% paraformaldehyde in 0.1 M phosphate buffer, pH 7.4 for 2 hours, washed with cacodylate (0.1 M, pH 7.4), post-fixed for 40 min in 1.0% osmium tetroxide, followed by 40 min wash in 1% uranyl acetate in water, dehydrated through increasing concentrations of alcohol and embedded in Durcupan ACM (Sigma Aldrich). Sections (50 nm thickness) contrasted with lead citrate and uranyl acetate were imaged on a Tecnai Spirit microscope with Eagle CCD camera (FEI Company).

### Cilia expression and beta cell proliferation

Neonatal rat islet monolayers were established during an initial 3-day culture period in complete neuronal medium with 11 mM glucose. To encourage cilia formation and growth, culture medium was replaced with serum and B-27-free neuronal medium containing 5.5 mM or 11 mM glucose for 36 hours. To induce cilia reabsorption and cell cycle progression following serum starvation, medium was replaced with complete neuronal medium containing 11 mM or 17 mM glucose and/or 15 μm ROCK inhibitor Y-27632 for 36 hours followed by fixation of cells in 4% EM-grade paraformaldehyde (PFA) for analysis.

### Statistical analyses

Means among three or more groups were compared by analysis of variance (ANOVA) in GraphPad Prism 6 software. If deemed significant, Tukey’s post-hoc pairwise comparisons were performed. Means between two groups were compared by Student *t* test. A confidence level of 95% was considered significant. Statistical significance of the data is indicated as follows: *P < 0.05, **P < 0.01, ***P < 0.001, ns no significant difference.

### Ethical approval

Animals were used under EPFL animal regulation guidelines and an IACUC approved protocol. Human islets were received from the University Hospital of Geneva and San Raffaele Scientific Institute, Milan through the ECIT islets for basic research program and were approved by the Institutional Review Board of the University Hospital of Geneva (CER Nr. 05-028) and by the Ethics Committee of the San Raffaele Scientific Institute of Milan (IPF002-2014). The University of Geneva and the San Raffaele Institute Ethics Committees waived the need for consent from the donors because islets were used for experimental research only when not suitable for clinical purposes and would otherwise have been destined for destruction. In such cases obtaining informed consent is not mandatory in Switzerland and Italy. Human pancreatic sections obtained via the nPOD tissue bank, University of Florida, Gainesville, FL, USA were harvested from cadaveric organ donors by certified organ procurement organizations partnering with nPOD in accordance with organ donation laws and regulations and classified as “Non-Human Subjects” by the University of Florida Institutional Review Board (IRB Nr. 392-2008) waiving the need for consent^66^,^67^. EPFL grants permit for the use of human material as long as the provider can certify that the samples were obtained according to local laws and regulations, as well as good practices in the country were they were collected.

### Data Availability. Associated Article

Cianciaruso, C. *et al*. Preparation of monolayer cultures of primary rat and human islet cells on glass for super-resolution and live cell imaging. Protocol Exchange doi: 1038/Protex.2017.039 (2017).

## Additional Information

**How to cite this article:** Phelps, E. A. *et al*. Advances in pancreatic islet monolayer culture on glass surfaces enable super-resolution microscopy and insights into beta cell ciliogenesis and proliferation. *Sci. Rep.* **7**, 45961; doi: 10.1038/srep45961 (2017).

**Publisher's note:** Springer Nature remains neutral with regard to jurisdictional claims in published maps and institutional affiliations.

## Supplementary Material

Supplementary Information

Supplementary Video S1

Supplementary Video S2

Supplementary Video S3
