# Corrigendum: Advances in pancreatic islet monolayer culture on glass surfaces enable super-resolution microscopy and insights into beta cell ciliogenesis and proliferation

**DOI:** 10.1038/srep46801

**Published:** 2017-05-09

**Authors:** Edward A. Phelps, Chiara Cianciaruso, Jaime Santo-Domingo, Miriella Pasquier, Gabriele Galliverti, Lorenzo Piemonti, Ekaterine Berishvili, Olivier Burri, Andreas Wiederkehr, Jeffrey A. Hubbell, Steinunn Baekkeskov

Scientific Reports 7: Article number: 4596110.1038/srep45961; published online: 04 12 2017; updated: 03 09 2017

This Article contains a typographical error in the Methods section under subheading ‘Data Availability. Associated Article’,

“Protocol Exchange doi: 1038/Protex.2017.039 (2017)”.

should read:

“Protocol Exchange doi: 10.1038/protex.2017.039”.

