# Supplementary material for: Advances in pancreatic islet monolayer culture on glass surfaces enable super-resolution microscopy and insights into beta cell ciliogenesis and proliferation
Source: Sci Rep. 2017 Apr 12;7:45961. doi: 10.1038/srep45961 (PMC5388888; doi:10.1038/srep45961)
Supplement: Supplementary Information [file srep45961-s1.pdf]

SUPPLEMENTARY INFORMATION

Advances in pancreatic islet monolayer culture on glass surfaces enable super-resolution microscopy and insights into beta cell ciliogenesis and proliferation

Edward A. Phelps, Chiara Cianciaruso, Jaime Santo-Domingo, Miriella Pasquier, Gabriele Galliverti, Lorenzo Piemonti, Ekaterine Berishvili, Olivier Burri, Andreas Wiederkehr, Jeffrey A. Hubbell, Steinunn Baekkeskov

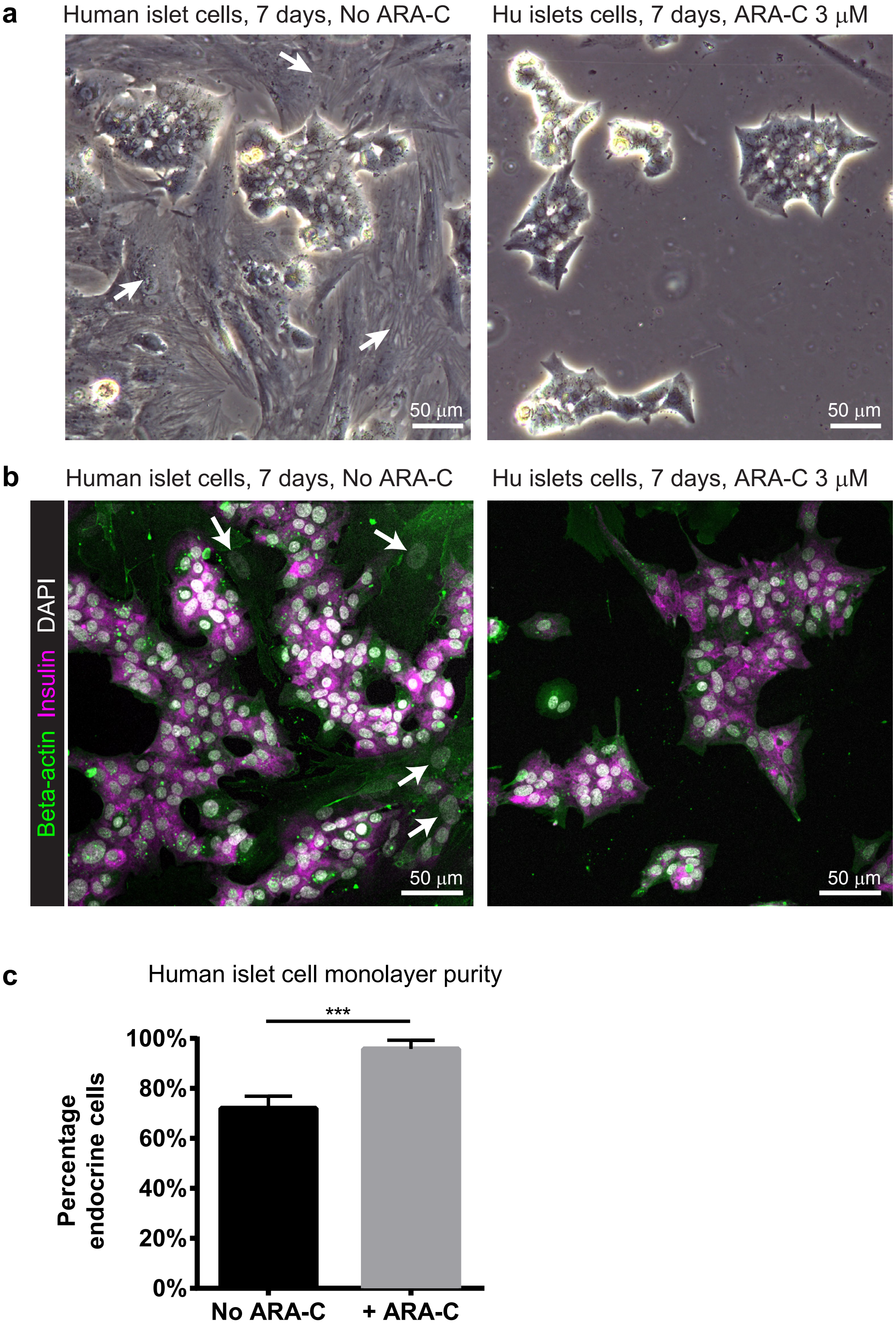

Figure S1. ARA-C-mediates elimination of fibroblast-like cells from human islet monolayers during culture for seven days on collagen IV-coated coverslips in neuronal medium. (a) Transmitted light images showing human islet cells on collagen IV-coated coverslips treated or not with ARA-C (3 μM). Arrows indicate regions of fibroblast-like cells. (b) Confocal images of human islet cells treated or not with ARA-C (3 μM) and immunostained for β-actin and insulin. Nuclei were stained with DAPI. Arrows indicate regions of fibroblast-like cells. (c) Quantification of islet cell purity. Beta cells, constituting the majority of endocrine cells in the culture were identified based on insulin expression, while non-beta endocrine cells were identified by characteristic morphology similar to beta cells, easily distinguished from the much larger fibroblast-like cells. Mean ± SEM (*n* = 6 random image fields per condition). Statistical analysis by Student's *t*-test (** $p < 0.01$).

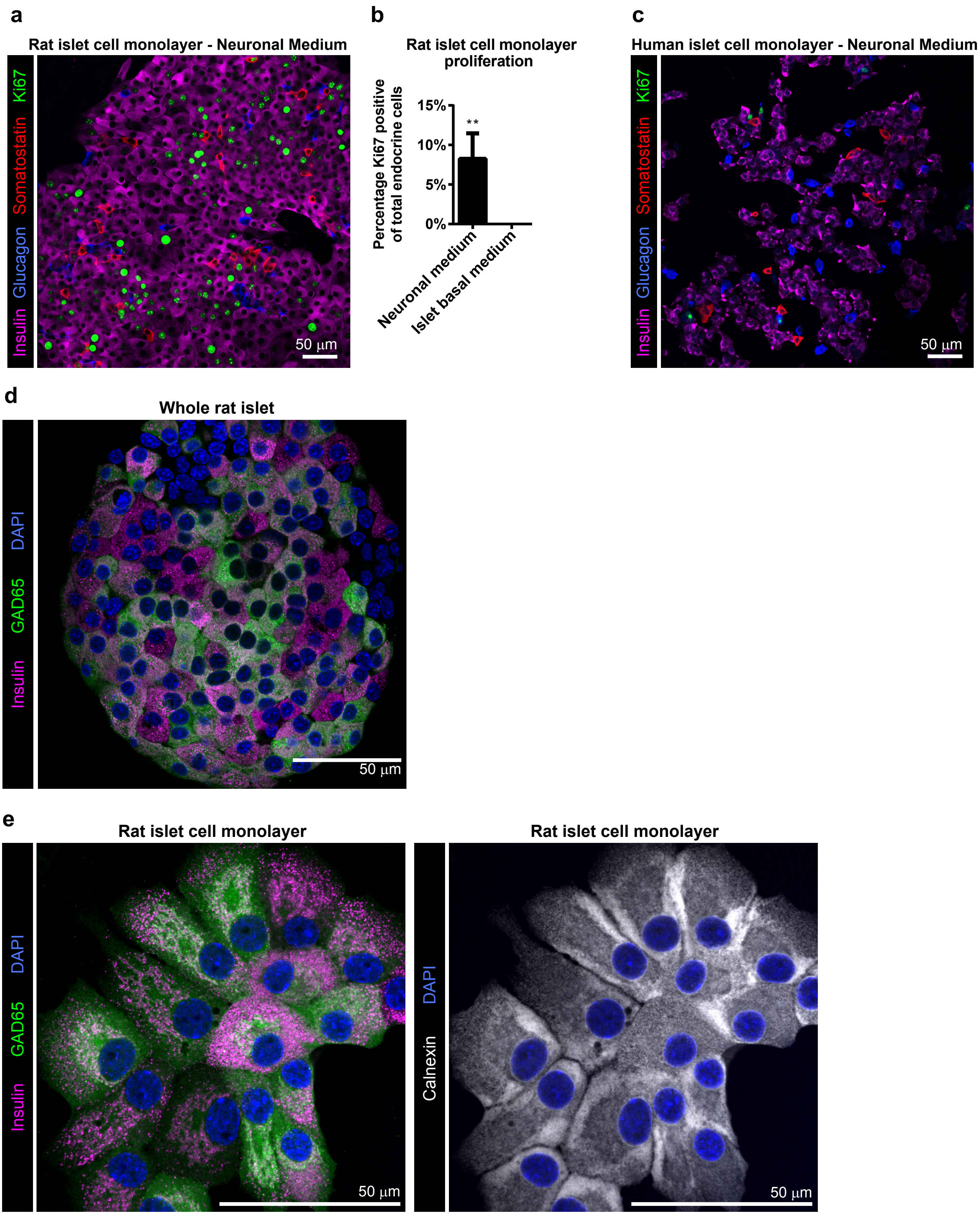

Figure S2. Islet cell monolayer proliferation. (a) A representative confocal image of rat islet cells cultured for seven days on laminin-coated coverslips in neuronal medium and immunostained for insulin, glucagon, somatostatin and Ki67. (b) Quantification of the percentage of Ki67+ rat islet cells when cultured in neuronal medium compared to islet basal medium. Mean ± SEM. (n = 10 random image fields). Statistical analysis by Student's t-test (*** $p < 0.001$). (c) A representative confocal image of adult human islet cells from an 18-year-old donor, cultured for seven days on collagen IV-coated coverslips in neuronal medium and immunostained for insulin, glucagon, somatostatin and Ki67. (d,e) Representative confocal microscopy images of (d) a whole rat islet and (e) a rat islet cell monolayer immunostained for insulin, the neuroendocrine protein GAD65, or calnexin. Nuclei are stained by DAPI.

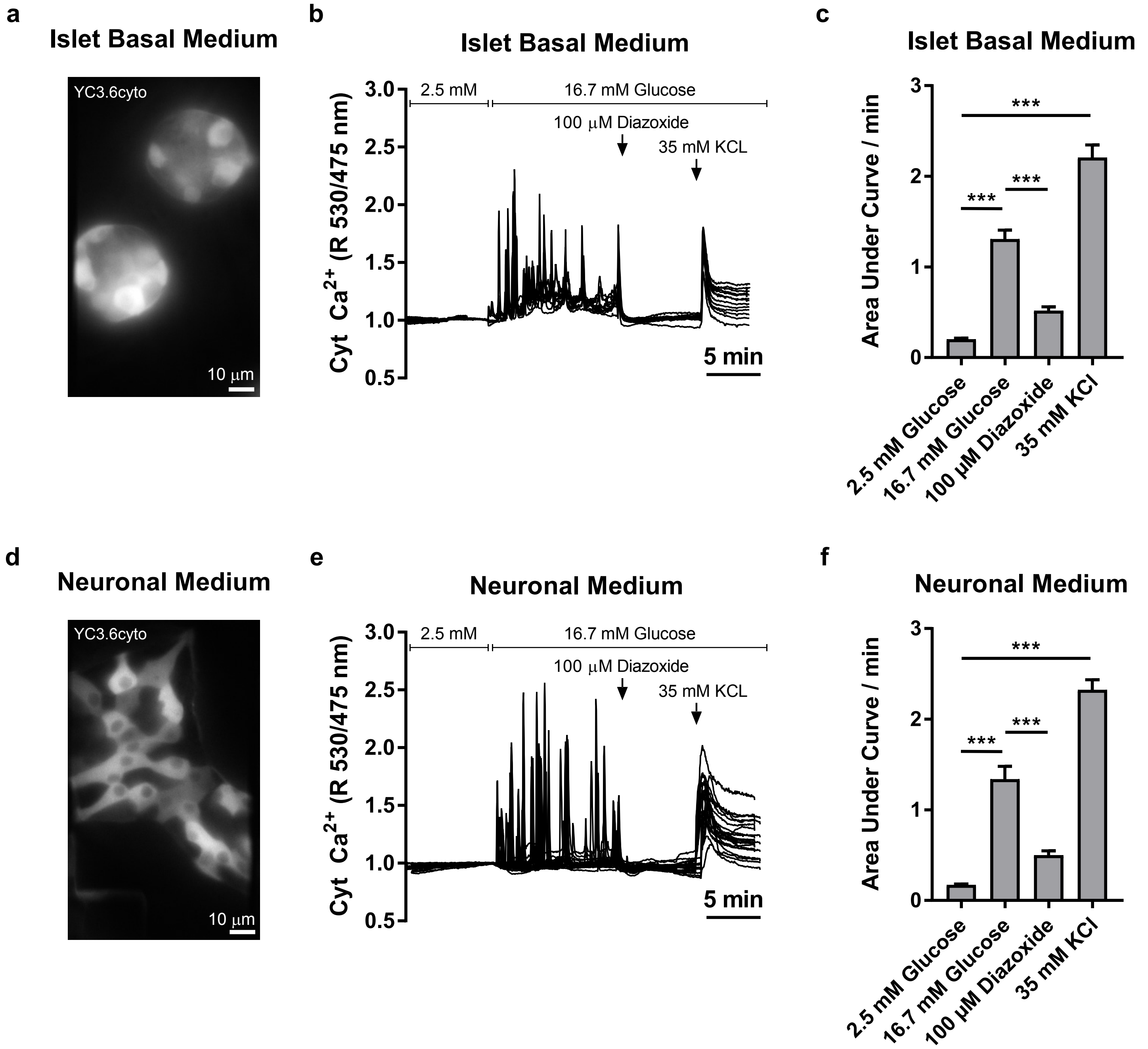

Figure S3. Comparison of calcium-responsive glucose sensing in rat islet cell monolayers cultured in islet basal medium (a-c) or neuronal medium (d-f). Cells were transduced with adenovirus to express Cameleon cytosolic Ca2+ sensor YC3.6cyto under the control of the rat insulin promoter. (a,d) Representative images of localization of YC3.6$_{cyto}$ in the cytosol of beta cells from islet cell monolayers cultured on laminin-coated glass in either (a) islet basal medium or (d) neuronal medium. (b,e) Representative Ca^{2+} imaging experiments in primary rat beta cells cultured on laminin-coated glass in either (b) islet basal medium or (e) neuronal medium, showing simultaneously-recorded Ca^{2+} traces from several beta-cells in a single field-of-view. (c,f) Quantification of the Ca^{2+} signaling during each phase of the experiment in primary rat beta cells cultured on laminin-coated glass in either (c) islet basal medium or (f) neuronal medium. Mean ± SD (n = 3 independent experiments, 9-26 cells analyzed per experiment). Statistical analysis by two-way ANOVA (*** $p < 0.001$, Tukey's post-hoc pairwise comparisons).

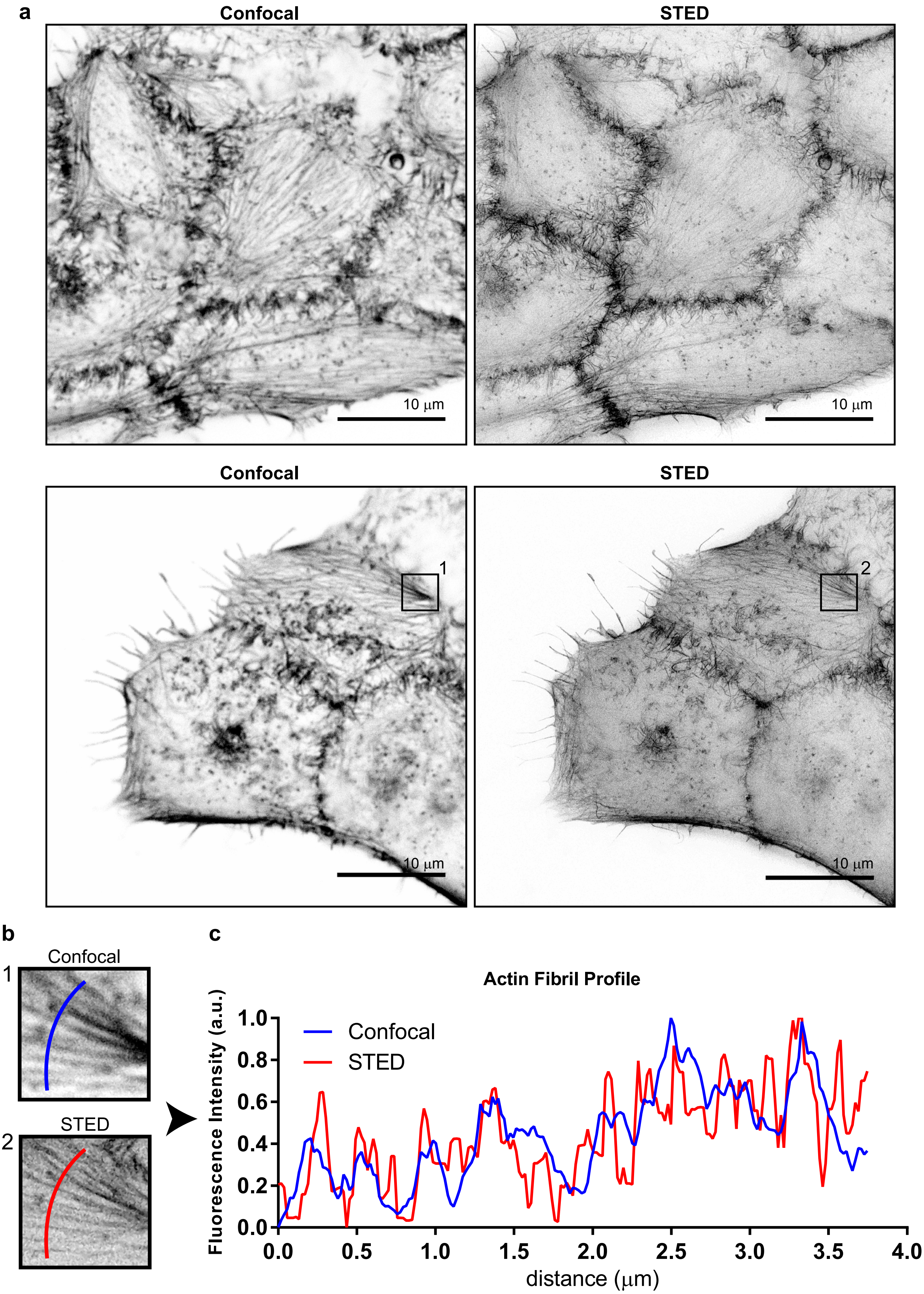

Figure S4. Confocal and STED super-resolution microscopy of the actin cytoskeleton in rat islet cell monolayers. (a) Representative images of rat islet cells stained with Alexa Fluor 488 phalloidin show high resolution structure of the beta cell cortical actin network. (b) Increased magnification of the bounded regions shown in the lower panels of (a). (c) Transverse fluorescence intensity profile measured across a group of actin filaments shown in (b) captured by STED and compared to confocal images.

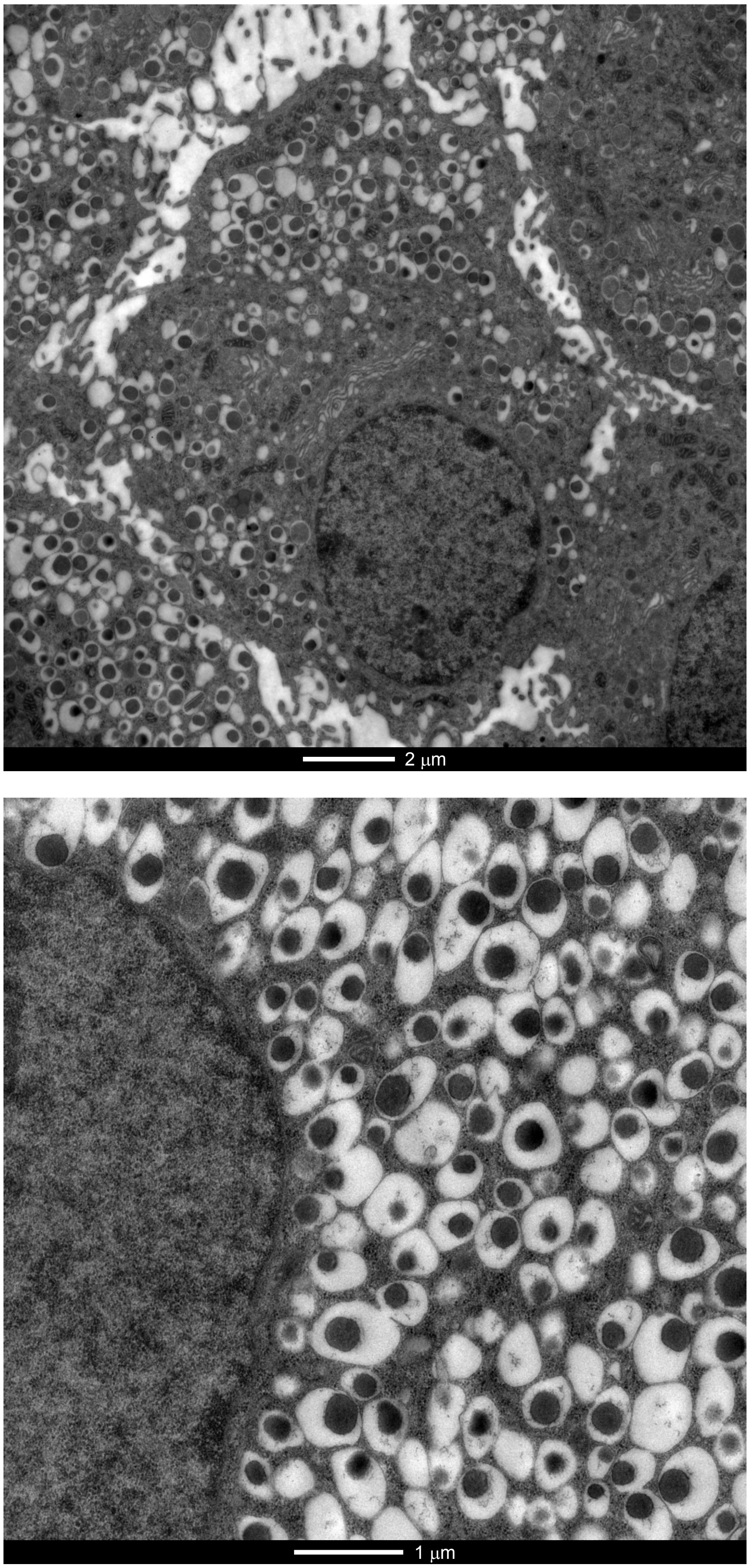

Figure S5. TEM of rat islets. Representative TEM images of whole rat islets used to measure the diameter of insulin granules in Figure 5C.

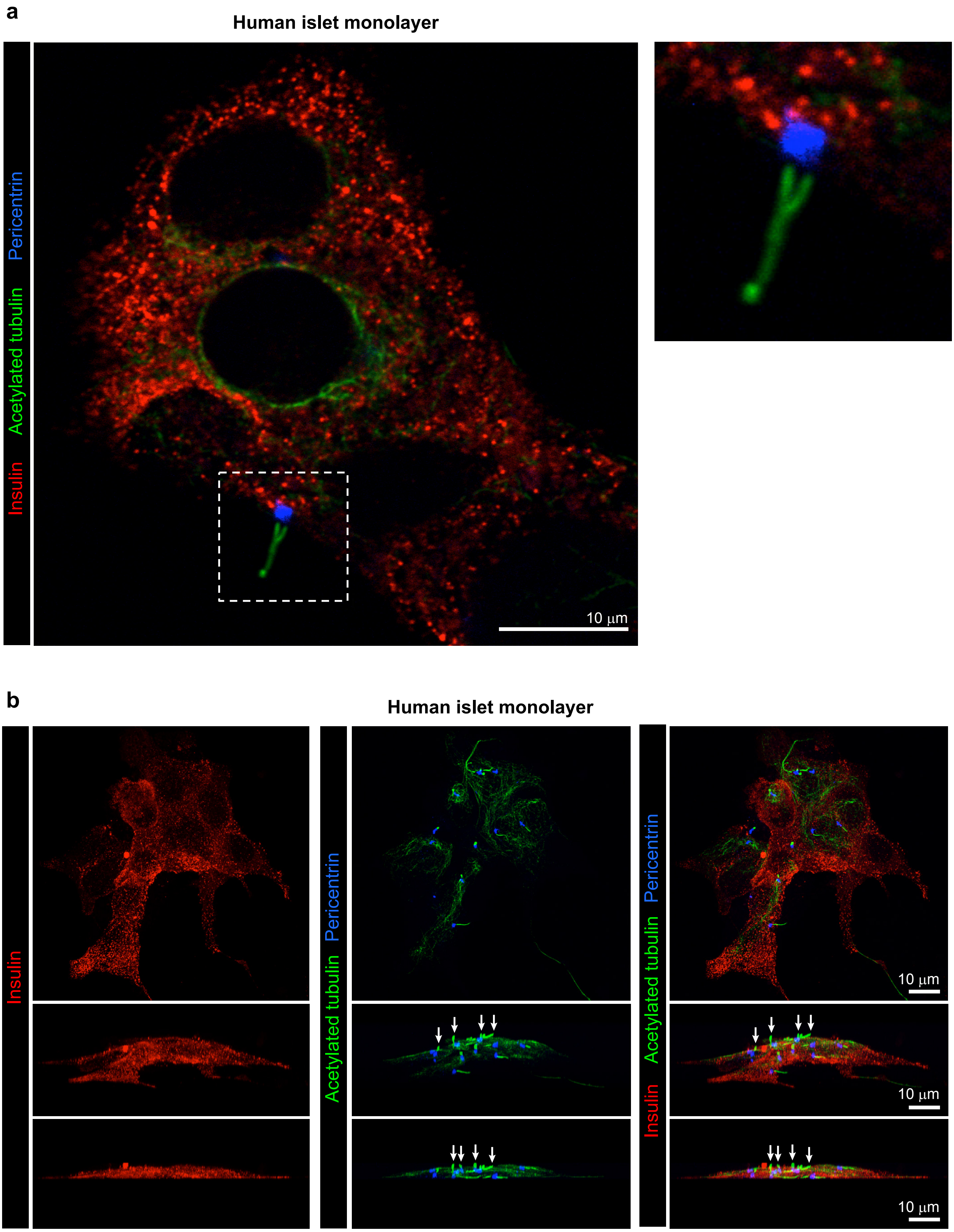

Figure S6. Confocal microscopy of primary cilia in human beta cells. (a) Left panel shows confocal analyses of primary human islet cells immunostained for insulin, the cilia marker acetylated alpha tubulin and the centrosomal protein pericentrin. Right panel, an increased magnification of the boxed region in the left panel, shows the localization of pericentrin at the base of a primary cilium originating from an insulin-expressing beta cell. (b) Three dimensional projections reconstructed from z-stacks obtained with confocal acquisitions of human islets cells immunostained for insulin, acetylated alpha tubulin and pericentrin. Arrows indicate individual cilia protruding from the apical side of human beta cells.

Rat islet cells in 5.5 mM glucose

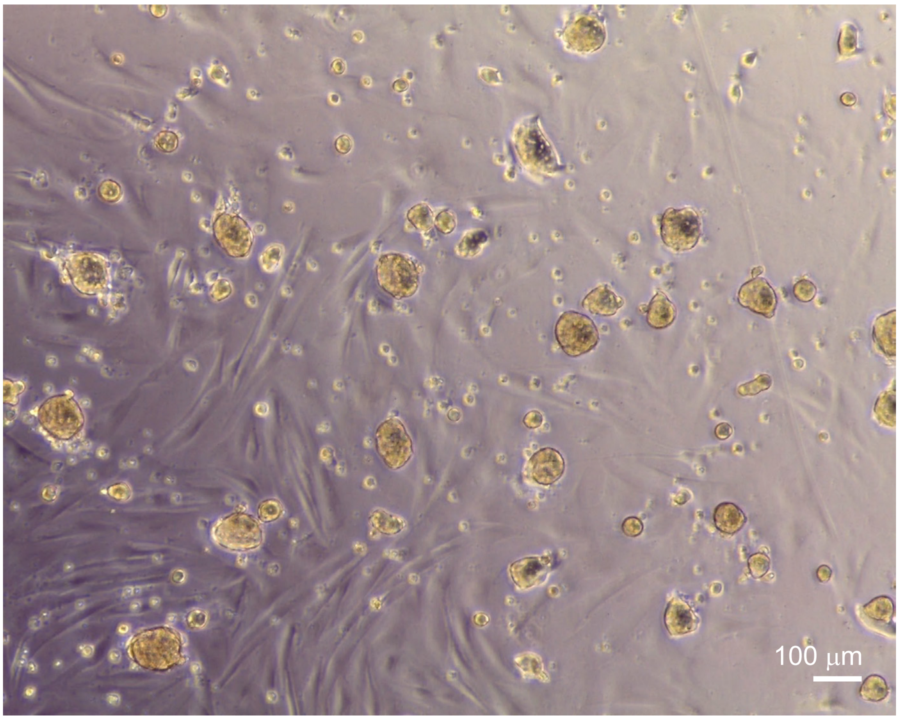

Rat islet cells in 11 mM glucose

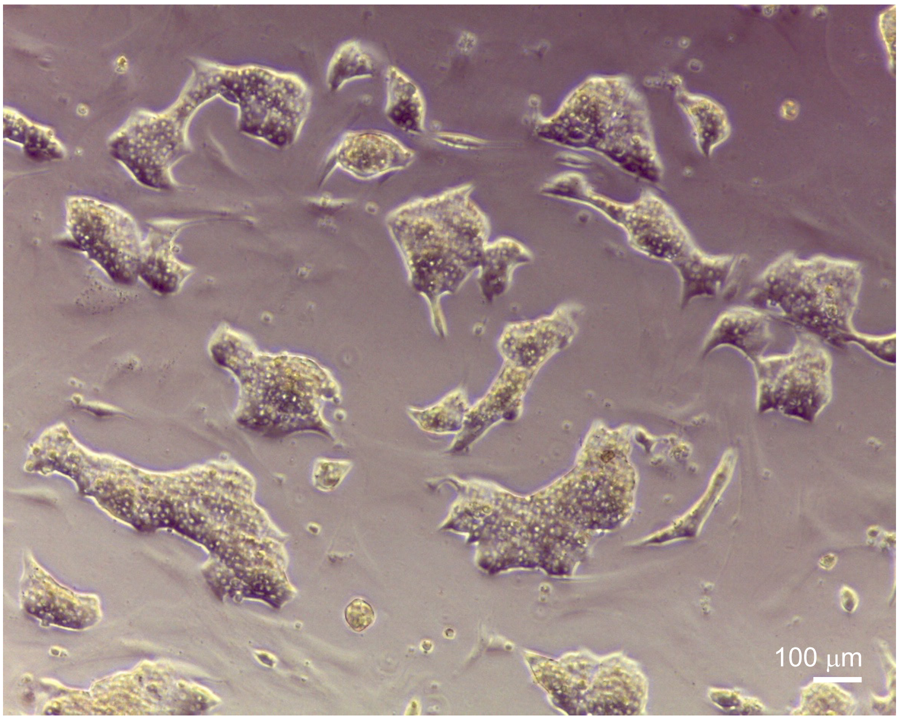

Figure S7. Glucose-dependent adhesion of rat islet cells. Representative transmitted light images of rat islet monolayer cells seeded on laminin coated glass for three days in neuronal medium containing 11 mM glucose and then switched to fresh neuronal medium containing either 5 mM or 11 mM glucose for 16 hours. The ability of cells to switch back and forth between rounded (5 mM glucose) vs a well spread (11 mM glucose) phenotype in response to a change in the glucose concentration was maintained for at least 3 days after the initial 3 day seeding period.

Video S1. Associated with Fig. 6. Time-lapse live-cell epifluorescence recording of rat islet beta cells expressing the genetically encoded ratiometric calcium indicator YC3.6$_{cyto,}$ during successive exposure to baseline glucose (2.5 mM glucose), high glucose stimulation (16.7 mM glucose), diazoxide inhibition of ATP-sensitive K+ channels, and complete depolarization (35 mM KCl).

Video S2. Associated with Supplementary Fig. S3. Time-lapse live-cell epifluorescence recording of rat islet beta cells cultured on laminin coated glass in islet basal medium expressing the genetically encoded ratiometric calcium indicator YC3.6$_{cyto,}$ during successive exposure to baseline glucose (2.5 mM glucose), high glucose stimulation (16.7 mM glucose), diazoxide inhibition of ATP-sensitive K+ channels, and complete depolarization (35 mM KCl).

Video S3. Associated with Supplementary Fig. S3. Time-lapse live-cell epifluorescence recording of rat islet beta cells cultured on laminin coated glass in neuronal medium expressing the genetically encoded ratiometric calcium indicator YC3.6$_{cyto,}$ during successive exposure to baseline glucose (2.5 mM glucose), high glucose stimulation (16.7 mM glucose), diazoxide inhibition of ATP-sensitive K+ channels, and complete depolarization (35 mM KCl).
